# Healthy Contact Lens Behaviors Communicated by Eye Care Providers and Recalled by Patients — United States, 2018

**DOI:** 10.15585/mmwr.mm6832a2

**Published:** 2019-08-16

**Authors:** Nuadum M. Konne, Sarah A. Collier, Jennifer Spangler, Jennifer R. Cope

**Affiliations:** ^1^Division of Foodborne, Waterborne, and Environmental Diseases, National Center for Emerging and Zoonotic Infectious Diseases, CDC; ^2^Research and Information Center, American Optometric Association, St. Louis, Missouri.

An estimated 45 million U.S. residents enjoy the benefits of contact lens wear, but many of them might be at increased risk for complications stemming from improper wear and care behaviors (*1*). One of the most serious complications of contact lens wear is a corneal infection known as microbial keratitis, which can sometimes result in reduced vision or blindness (*2*). In 2014, 50% of contact lens wearers reported ever sleeping in contact lenses, and 55% reported topping off* their contact lens solutions (*3*), which put them at greater risk for a contact lens–related eye infection (*2*,*4*). Data on communication between eye care providers and contact lens wearers on contact lens wear and care recommendations are limited. Two surveys were conducted to better understand and assess contact lens education about nine recommendations: the first assessed contact lens wearer experiences regarding recommendations received from eye care providers during their most recent appointment; the second evaluated provider-reported practices for communicating contact lens wear and care recommendations to their patients. One third (32.9%) of contact lens wearers aged ≥18 years recalled never hearing any lens wear and care recommendations. Fewer than half (47.9%) recalled hearing their provider recommend not sleeping in lenses at their last visit, and 19.8% recalled being told to avoid topping off their contact lens solution. A majority of providers reported sharing recommendations always or most of the time at initial visits, regular checkups, and complication-related visits. Providers reported sharing nearly all recommendations more frequently at initial and complication-related visits than at regular checkups. Of the nine recommendations for safe contact lens wear and care, eye care providers at regular checkups most often recommend complying with the recommended lens replacement schedules (85% of regular visits), not sleeping in lenses (79.0% of regular visits), and not topping off solutions (64.4% of regular visits). Eye care providers play an important role in the health of their contact lens–wearing patients and can share health communication messages with their patients to help educate them about healthy wear and care habits. These findings can assist in the creation of health communication messages to help encourage eye care providers to communicate more effectively with their patients.

The Porter Novelli 2018 summer HealthStyles survey, an online survey, was used to estimate the number of contact lens wearers who reported receiving contact lens wear and care recommendations from their eye care provider during their most recent visit. The following nine recommendations were evaluated: 1) avoid sleeping overnight or napping in lenses, 2) wash and dry hands before inserting or removing lenses, 3) replace lenses as often as recommended, 4) replace lens case at least once every 3 months, 5) avoid storing lenses in water, 6) avoid rinsing lenses in water, 7) avoid topping off solution, 8) avoid swimming in lenses, and 9) avoid showering in lenses. The Internet survey included 4,088 participants^†^ who are part of market research firm GfK’s Knowledge Panel. Panel members are recruited using address-based probability sampling methods and provided with Internet access and a computer if needed. Statistical weighting was employed to make the panel representative of the U.S. population by race, ethnicity, sex, age, household income, household size, education level, census region, metropolitan status, and Internet access before joining the panel. Respondents received 5,000 cash-equivalent reward points worth approximately $5. 

To describe provider health communication practices, 1,100 randomly selected (based on geographic region of current practice) licensed, practicing eye care providers were surveyed. The survey was piloted by members of the American Optometric Association’s Contact Lens and Corneal section, and changes were made based on feedback from members. Invitations to participate in the survey were e-mailed by the American Optometric Association, primarily to optometrists working in private practice settings for ≥5 years. Four reminder e-mails were sent, one every other week, and the survey was officially closed after 2 months. Of the 1,100 providers who were sent the survey, 365 (33%) responded. Survey questions assessed how often providers mentioned the same nine contact lens wear and care recommendations to their patients at initial contact lens fittings, during regular checkups and annual visits, and at visits when patients are seen for contact lens–related complications. 

Frequencies for both surveys were calculated using SAS (version 9.4; SAS Institute) with complex sample survey procedures when appropriate. To protect participant confidentiality, no individual identifiers were included in the data set received by investigators. As a result, analyses of data from the 2018 HealthStyles Fall survey were declared exempt by CDC’s institutional review board. Because no interaction or intervention with human subjects occurred by U.S. Department of Health and Human Services researchers for the provider survey and no personally identifiable information was used, collected, or transmitted during the course of this analysis of previously collected data, this analysis was not considered human subjects research^§^ requiring review by CDC’s institutional review board.

The majority of contact lens–wearing patients surveyed reported wearing soft contact lenses, were non-Hispanic (85.8%), white (77.7%), and female (59.2%) (Table 1). One third (32.9%) of contact lens wearers recalled never hearing any lens wear and care recommendations (Table 2). Fewer than half of patients recalled hearing each of the nine recommendations. During their most recent visit, patients most frequently recalled hearing their provider recommend not sleeping in lenses (47.9%), washing and drying hands before inserting or removing lenses (46.9%), and complying with lens replacement schedules (41.6%), and least frequently recalled being told to avoid swimming (12.4%) and showering in their lenses (8.3%). A majority (54.7%–97.4%) of providers reported sharing all nine messages always or most of the time at initial visits, regular checkups, and complication-related visits (Table 2). Providers reported sharing messages more frequently at initial visits and complication-related visits than at regular checkups. At regular visits, of the nine recommendations, eye care providers reported most often recommending complying with lens replacement schedules (85% of regular visits), washing and drying hands before inserting or removing lenses (79%), and not sleeping in lenses (79%), and least often recommended not swimming (63%) or showering in lenses (55%).

**TABLE 1:** Characteristics of contact lens–wearing patients, (N = 733) — Porter Novelli HealthStyles Internet survey, United States, 2018

Characteristic	Weighted no. (%*)
**Type of contacts worn**	
Soft	629 (85.8)
Rigid/Gas permeable	55 (7.5)
Orthokeratology	5 (0.7)
Other^†^	50 (6.9)
**Gender**	
Female	434 (59.2)
Male	299 (40.8)
**Age group (yrs)**	
18–24	116 (15.8)
25–34	177 (24.2)
35–44	174 (23.7)
45–54	127 (17.4)
55–64	88 (12.0)
65–74	42 (5.7)
≥75	9 (1.3)
**Race**	
White	569 (77.7)
Black/African-American	72 (9.9)
Asian	68 (9.2)
American Indian or Alaskan Native	7 (0.9)
Hawaiian/Pacific Islander	3 (0.4)
Multiracial	14 (2)
**Hispanic ethnicity**	
Hispanic	108 (14.8)
Non-Hispanic	624 (85.2)
**Education**	
Less than high school	34 (4.6)
High school	188 (25.7)
Some college	194 (26.4)
Bachelor's degree or higher	317 (43.3)

* Some categories do not sum to 100 because of rounding.

^†^ A type of contact lens not included among the survey choices.

**TABLE 2:** Percentage of contact lens–wearing patients (N = 733) who recalled hearing their eye care provider mention the recommendations,* and percentage of eye care providers (N = 365) who reported making contact lens wear and care recommendations to their contact lens–wearing patients always or most of the time^†^ — United States, 2018

Recommendation	Patients % (95% CI)	Providers % (95% CI)		
	Most recent visit	Initial fittings	Regular checkups	Contact lens–related complication visit
Avoid sleeping overnight or napping in lenses	47.9 (43.6–52.2)	96.8 (94.8–98.8)	79.0 (74.5–83.6)	97.4 (95.6–99.2)
Wash and dry hands before inserting or removing lenses	46.9 (42.6–51.2)	97.1 (95.2–99.0)	79.0 (74.4–83.5)	92.2 (89.1–95.2)
Replace lenses as often as recommended	41.6 (37.4–45.7)	96.1 (94–98.3)	85.1 (81.1–89.1)	97.4 (95.6–99.2)
Replace lens case at least every 3 months	23.8 (20.1–27.5)	83.2 (79.0–87.4)	62.6 (57.2–68.0)	86.0 (82.0–89.9)
Avoid storing lenses in water	21.0 (17.4–24.7)	92.6 (89.6–95.5)	70.4 (65.2–75.5)	86.3 (82.5–90.2)
Avoid rinsing lenses in water	19.8 (16.3–23.4)	90.3 (87.0–93.6)	70.7 (65.6–75.8)	86.9 (83.1–90.7)
Avoid “topping off” solution	19.8 (16.3–23.3)	91.3 (88.1–94.4)	64.4 (59.0–69.8)	89.9 (86.5–93.3)
Avoid swimming in lenses	12.4 (9.4–15.4)	83.9 (79.8–88.0)	63.0 (57.6–68.4)	81.7 (77.3–86.1)
Avoid showering in lenses	8.3 (5.8–10.8)	73.2 (68.3–78.2)	54.7 (49.1–60.3)	77.8 (73.1–82.5)
Heard or stated all of recommendations	3.6 (1.7–5.4)	57.1 (51.9–62.2)	39.6 (34.5–44.7)	61.5 (56.5–66.5)
Heard or stated none of recommendations	32.9 (28.8–36.9)	1.4 (0.20–2.6)	1.4 (0.20–2.6)	0.60 (0.00–1.3)

**Abbreviation:** CI = confidence interval.

* Based on responses to Porter Novelli HealthStyles survey.

^†^ Based on responses to American Optometric Association survey.

## Discussion

Eye care providers report mentioning nine contact lens wear and care recommendations to patients frequently, but patients recall hearing these messages less often. This discrepancy in provider-patient communication has been reported previously in many medical specialties, despite the identification of patient communication as an important physician competency, and an important component of medical education curricula for physicians in the United States (*5*). Effective communication between physicians and patients can have a positive impact on health (*6*). Studies demonstrate evidence of positive associations between physician communication behaviors and positive patient outcomes (*5*,*7*). Patients continue to report that many of their informational needs remain unmet during a doctor’s visit (*6*). The gap between what providers say and what patients hear might be a factor in the large proportion of contact lens wearers reporting behaviors that put them at risk for a contact lens–related eye infection (*1*,*3*). Addressing this gap might improve contact lens wear and care practices.

These survey results show that eye care providers most often recommend complying with the recommended lens replacement schedules. However, other risk behaviors are more common or pose a greater risk for contact lens–related eye infections. For example, sleeping in contact lenses and topping off lens solution both increase the risk for contact lens–related eye infections approximately sixfold (*2*,*4*), and both behaviors have been reported by a majority of lens wearers (*3*). Storing lenses in water increases the risk for infection up to sixteenfold because microorganisms living in water can be transferred to the eye (*8*). Even household tap water, although treated to be safe for drinking, is not sterile and contains microorganisms that can contaminate lens cases and contact lenses and cause eye infections. Given the evidence showing that sleeping in lenses, topping off solution, and exposing lenses directly to water are risky behaviors and the limited time allowed for a visit with an eye care provider, providers might consider prioritizing these recommendations over others during all types of interactions with contact lens wearers. To alleviate the time constraints of a typical visit, providers can also provide communication materials, like CDC’s tear off pads, for their patients to take home.

The findings in this report are subject to at least three limitations. First, the results are taken from two different surveys; therefore, the patients surveyed are likely not the patients of the surveyed eye care providers, and direct connections cannot be established. Second, the provider survey had a response rate of 33%, which, although not necessarily unexpected for a survey of practicing providers, might limit the representativeness of the survey and might introduce bias. Systematic differences between eye care providers who completed the survey and those who did not could not be assessed. Finally, although the results of the two surveys demonstrate an apparent gap in patient-provider communications, they do not identify other variables that might explain the reason for this gap. Respondents to the patient survey were not asked to describe their health literacy, which is an equally important factor and component of adherence to care recommendations. In the United States, 12% of adults have proficient health literacy, which suggests that nearly 90% of U.S. residents might find it challenging to obtain, process, and understand basic health information and services needed to make decisions about their health (*9*). Therefore, identifying ways to promote healthy contact lens practices among patients with low health literacy is challenging.

Previous studies have identified health behaviors that can reduce the risk for contact lens–associated eye infections (e.g., not sleeping in lenses, not exposing lenses to water, and using fresh disinfecting solution to store lenses) (*2*,*4*). Although eye care providers report mentioning these behaviors to their patients frequently, patients report hearing the messages less frequently, suggesting that new communication strategies might be needed. CDC has developed health communication materials^¶^ that target contact lens wearers and eye care providers. Eye care providers can obtain these materials to share with their patients to help educate them about healthy wear and care habits. Eye care provider communication techniques to inform patients of health risks that are easy to understand, specific, use repetition, minimize jargon, and checked for patient’s understanding of the information presented are most likely to be effective (*5*). Improving communications between providers and patients could help contact lens wearers understand proper eye care (*10*).

**Summary**

**What is already known about this topic?**

Most of the 45 million contact lens wearers in the United States practice at least some behaviors that put them at risk for serious eye infections.

**What is added by this report?**

Surveys of contact lens wearers and eye care providers were conducted in 2018. One third of lens wearers recalled never hearing any lens care recommendations. Most eye care providers reported sharing recommendations always or most of the time.

**What are the implications for public health practice?**

Eye care providers play an important role in the health of their contact lens–wearing patients and can share health communication messages with their patients to help educate them about healthy wear and care habits.

## Footnotes

* Adding new solution to existing solution in the contact lens case instead of emptying and cleaning the case before adding new solution.

† Porter Novelli Public Services. Summer HealthStyles 2018 methodology; Washington, DC.

§ As defined in 45 CFR part 46. https://www.hhs.gov/ohrp/regulations-and-policy/regulations/45-cfr-46/index.html.

¶ https://wwwn.cdc.gov/pubs/cdcinfoondemand.aspx?ProgramID=192.
