# *Erratum*: Vol. 68, No. 32

**DOI:** 10.15585/mmwr.mm6837a4

**Published:** 2019-09-20

In the report “Healthy Contact Lens Behaviors Communicated by Eye Care Providers and Recalled by Patients — United States, 2018,” on page 695, the first sentence of the third paragraph should have read “The majority of contact lens–wearing patients surveyed reported wearing soft contact lenses (**85.8%**). **In addition, the majority of patients surveyed were non-Hispanic** (**85.2%**), white (77.7%), and female (59.2%) (Table 1).”

